# Case–Control Study of an Acute Aflatoxicosis Outbreak, Kenya, 2004

**DOI:** 10.1289/ehp.8384

**Published:** 2005-08-09

**Authors:** Eduardo Azziz-Baumgartner, Kimberly Lindblade, Karen Gieseker, Helen Schurz Rogers, Stephanie Kieszak, Henry Njapau, Rosemary Schleicher, Leslie F. McCoy, Ambrose Misore, Kevin DeCock, Carol Rubin, Laurence Slutsker, the Aflatoxin Investigative Group

**Affiliations:** 1 National Center for Environmental Health,; 2 National Center for Infectious Diseases, and; 3 Epidemiology Program Office, Centers for Disease Control and Prevention, Atlanta, Georgia, USA; 4 Food and Drug Administration, Washington, DC, USA; 5 Preventive and Promotive Health Services, Kenya Ministry of Health, Nairobi, Kenya; 6 Centers for Disease Control and Prevention, Kenya Field Office, Nairobi, Kenya; 7 Centers for Disease Control and Prevention, Kenya Field Office, Kisumu, Kenya

**Keywords:** albumin adducts, aflatoxicosis, aflatoxin, Kenya, lysine, maize

## Abstract

Objectives: During January–June 2004, an aflatoxicosis outbreak in eastern Kenya resulted in 317 cases and 125 deaths. We conducted a case–control study to identify risk factors for contamination of implicated maize and, for the first time, quantitated biomarkers associated with acute aflatoxicosis.

Design: We administered questionnaires regarding maize storage and consumption and obtained maize and blood samples from participants.

Participants: We recruited 40 case-patients with aflatoxicosis and 80 randomly selected controls to participate in this study.

Evaluations/Measurements: We analyzed maize for total aflatoxins and serum for aflatoxin B_1_–lysine albumin adducts and hepatitis B surface antigen. We used regression and survival analyses to explore the relationship between aflatoxins, maize consumption, hepatitis B surface antigen, and case status.

Results: Homegrown (not commercial) maize kernels from case households had higher concentrations of aflatoxins than did kernels from control households [geometric mean (GM) = 354.53 ppb vs. 44.14 ppb; *p* = 0.04]. Serum adduct concentrations were associated with time from jaundice to death [adjusted hazard ratio = 1.3; 95% confidence interval (CI), 1.04–1.6]. Case patients had positive hepatitis B titers [odds ratio (OR) = 9.8; 95% CI, 1.5–63.1] more often than controls. Case patients stored wet maize (OR = 3.5; 95% CI, 1.2–10.3) inside their homes (OR = 12.0; 95% CI, 1.5–95.7) rather than in granaries more often than did controls.

Conclusion: Aflatoxin concentrations in maize, serum aflatoxin B_1_–lysine adduct concentrations, and positive hepatitis B surface antigen titers were all associated with case status.

Relevance: The novel methods and risk factors described may help health officials prevent future outbreaks of aflatoxicosis.

During January–June 2004, the Kenya Ministry of Health (MOH) and partners identified 317 cases of acute hepatic failure in eastern Kenya; 125 cases occurred in persons who subsequently died during the illness. Seven patients had serum samples analyzed at the Kenya Medical Research Institute (KEMRI), and all were negative for viruses known to cause hepatic disease in Kenya (e.g., yellow fever; Rift Valley fever; dengue; acute hepatitis A, B, and C; West Nile virus; and Chikungunya and Bunyamwera) (American Public Health Association 2000). Because aflatoxicosis outbreaks had occurred previously in that geographical area, the MOH suspected that the unusually high number of patients with acute hepatic failure might have acquired aflatoxicosis from eating contaminated maize (Ngindu et al. 1982). Public health officials sampled maize from the affected area and found concentrations of aflatoxin B_1_ as high as 4,400 ppb, which is 220 times greater than the 20 ppb limit for food suggested by Kenyan authorities (Onsongo 2004). Although aflatoxicosis outbreaks have occurred periodically in Africa and Asia, this outbreak resulted in the largest number of fatalities ever documented (Krishnamachari et al. 1975a, 1975b; Lye et al. 1995).

Aflatoxins are produced by *Aspergillus* spp. fungi that grow on a wide variety of grains and nuts (Patten 1981). The human gastrointestinal tract rapidly absorbs aflatoxins after consumption of contaminated food, and the circulatory system transports the aflatoxins to the liver (Fung and Clark 2004). From 1 to 3% of ingested aflatoxins irreversibly bind to proteins and DNA bases to form adducts such as aflatoxin B_1_–lysine in albumin (Skipper and Tannenbaum 1990). Disruption of proteins and DNA bases in hepatocytes causes liver toxicity (Tandon et al. 1978).

Early symptoms of hepatotoxicity from aflatoxicosis can manifest as anorexia, malaise, and low-grade fever. Aflatoxicosis can progress to potentially lethal acute hepatitis with vomiting, abdominal pain, hepatitis, and death (Etzel 2002). Because aflatoxin B_1_–lysine adducts are not repaired, their half-life in human serum is approximately 20–60 days (i.e., similar to that of unbound albumin) (McCoy L, personal communication; Sabbioni et al. 1987).

Information about risk factors associated with outbreaks of aflatoxicosis is limited. In addition, only a few animal studies have measured aflatoxin concentrations because unbound aflatoxins remain in the blood for a very short period of time after exposure (i.e., 13–120 min) (Unger et al. 1977; Wong and Hsiech 1978). The primary objective of our case–control study was to identify risk factors for acute aflatoxicosis. The secondary objective was to determine the concentrations of aflatoxin in maize, bound aflatoxin in serum, and hepatitis B surface antigen associated with acute aflatoxicosis.

## Materials and Methods

### Selection of case patients.

To focus the investigation on typical cases of presumed aflatoxicosis, our case definition was restricted to acute jaundice of unknown origin (i.e., no history of cirrhosis or obstructive liver disease) leading to hospitalization, during the peak of the epidemic, in the areas most affected by the outbreak. This case definition was based on information gathered by a descriptive epidemiology investigation conducted by the MOH and partners in May 2004. The descriptive epidemiology investigation found that a large number of patients with presumed aflatoxicosis had sought treatment at Makindu Sub-District Hospital (Makueni District) during 18 May–7 June and at Mutomo Mission Hospital (Kitui Districts) during 28 May–9 June. We did not restrict cases to live case patients or to case patients from which KEMRI had obtained blood samples because we did not want to introduce bias in our assessment of risk factors associated with disease.

To select 40 patients that met our case definition, we reviewed hospital records for the relevant time period and identified 19 case patients admitted to Makindu Sub-District Hospital and 21 case patients admitted to Mutomo Mission Hospital. All of the 29 case patients were alive at the time of the investigation, and all of the families of 11 deceased case patients verbally consented to participate in the study.

### Selection of controls.

We randomly selected two controls from each case patient’s village because the descriptive epidemiology investigation suggested that these individuals would share similar soil, microclimate, and farming practices. Because the descriptive epidemiology investigation did not find a significant association among sex, case status, and case fatality, we did not match cases and controls by sex. To choose each control, we spun a bottle in front of the village elder’s home and walked to the fifth house in the direction indicated by the bottle (or to the third house in sparsely populated areas). At the selected household, we identified all residents who had slept in the house the night before, and we used a random number list to select one of these household residents. We excluded infants who were solely breast-feeding because they would not have been directly exposed to aflatoxin B_1_ found in maize. If selected individuals were not at their homes, we attempted to reach them wherever they were. All controls verbally consented to participate in the study.

### Survey instrument.

A literature review and the descriptive epidemiology investigation allowed development of hypotheses about the relationship between aflatoxicosis and methods of handling maize. We developed a questionnaire to elicit information about maize and protein consumption, the quality of home-grown and purchased maize products, maize storage and cooking practices, and associated illness and death of family members and pet dogs. All questions related to the relevant exposure period, which was designated as 1 month before the onset of case patients’ illness or 1 month before controls heard about the outbreak.

Teams piloted the questionnaire on hospitalized patients who had presumed aflatoxicosis in Thika District. Local public health officials translated the questionnaire, which was written in English, into Kikamba and Kiswahili as needed. Teams carried measuring cups to obtain standardized information on maize food portions consumed by participants.

### Food sample collection.

We obtained samples of maize products from participants to quantify personal exposure to aflatoxins. We collected samples from case households if they had maize in storage from the month before individuals developed aflatoxicosis (median date of symptom onset, 20 May 2004). We collected samples from control households if they had maize in storage from the month before hearing about the outbreak (median date of first hearing about the outbreak, 19 May 2004). We used metal cups to obtain multiple samples from different areas of the maize containers. These samples were combined in a paper bag to obtain 1 kg of maize for analysis. Collected maize products were replaced with commercial maize meal.

### Blood sample collection.

We obtained blood samples from participants to quantify their exposure to aflatoxins in the preceding month. With the exception of six case-patients from whom KEMRI had banked blood in May, we collected approximately 5–10 mL of venous blood in a Vacutainer tube with gel separators from all participants. All blood samples were transported on ice to KEMRI for serum separation.

### Laboratory analysis.

We analyzed maize samples using the VICAM AflaTest (VICAM, Watertown, MA, USA) immunoaffinity fluorometric method that quantitated total aflatoxin concentrations. Ground maize (50 g) that passed through a no. 20 sieve was mixed with 100 mL of a methanol:water mixture (80:20) with 5 g sodium chloride. The twice-filtered mixture (2 mL) was then passed through the immunoaffinity column at a rate of 1–2 drops/sec. The columns were washed with water, and the aflatoxins were recovered using 1 mL methanol. The methanol extract was read using a calibrated Vicam Series-4 Fluorometer set at 360 nm excitation and 450 nm emission. This method had an afla-toxin recovery of ≥85% and a detection limit of 1 ppb (VICAM 2001).

The Centers for Disease Control and Prevention (CDC) analyzed the serum specimens for aflatoxin B_1_–lysine albumin adducts using high-performance liquid chromatography (HPLC) and isotope dilution tandem mass spectrometry (McCoy et al. 2005). After enzymatic hydrolysis of serum albumin, aflatoxin B_1_–lysine adducts were extracted using solid-phase cartridges and separated using isocratic reversed-phase chromatography. We used positive ion electrospray with selected reaction monitoring mass spectrometry to measure aflatoxin B_1_–lysine adducts and its corresponding D4-labeled internal standard. We measured total serum albumin using a bromocresol purple binding assay and a microplate reader. The limit of detection of aflatoxin B_1_–lysine albumin adducts was 0.0003 ng/mg. The CDC also analyzed all remaining sera for hepatitis B surface antigen using ETI-MAK-2 PLUS enzyme immunoassay kits from DiaSorin (DiaSorin, Stillwater, MN).

### Data management and analysis.

Data were analyzed using SAS, version 8.02 (SAS Institute, Cary, NC). We used conditional logistic regression to calculate odds ratios (ORs) between case status and participants’ methods of harvesting, storing, and preparing maize. We also used conditional logistic regression models to explore the relationship between case status, maize and protein consumption, aflatoxin concentrations in maize, aflatoxin B_1_–lysine adduct concentrations, and hepatitis B surface antigen titers in serum. We restricted mixed linear regression models to controls because we wanted to investigate the relationship between serum aflatoxin concentrations and methods of harvesting, storing, and preparing maize, daily maize and protein consumption, and total aflatoxin concentrations in maize using a sample that more closely resembled the general population. We also used Cox proportional hazards models to explore the relationship between the number of days case patients survived after the onset of jaundice and aflatoxin concentrations in maize, aflatoxin B_1_–lysine adducts concentrations in serum, hepatitis B surface antigen titers, and reported maize and protein consumption. Calculations were adjusted for age, sex, and participant’s district.

## Results

### Demographic information.

With few exceptions, case patients (*n* = 40) and controls (*n* = 80) had similar demographic characteristics (Table 1). Half of the participants lived in the Makueni District and the other half lived in the Kitui District. The mean age of case patients was similar to that of controls [22.5 years (range, 1.3–80.0 years) vs. 26 years (range, 0.5–75.0 years), respectively]. When compared with controls, more of the case patients were male (62.5% vs. 33.8%, respectively; *p* = 0.003). Case patients were also more likely than controls to report having family members with acute jaundice during the 2 months before the study (37.5% vs. 3.8%; *p* < 0.001). As of 9 August, 18 of the 40 case patients (7 additional case patients since completion of our study) had died of acute liver failure.

### Food consumption and maize aflatoxin analysis.

**Figure 1:**
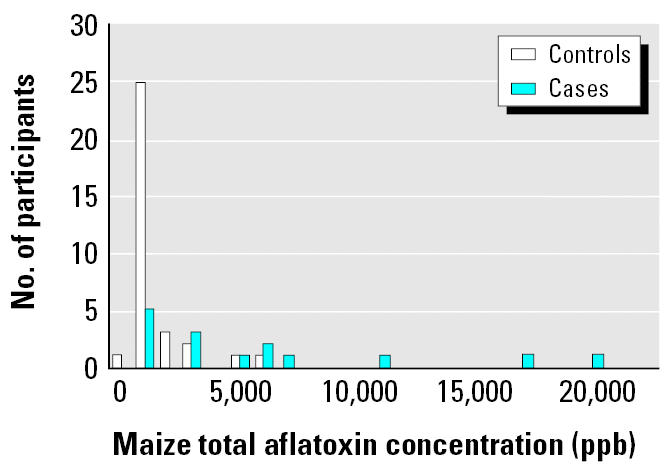
Frequency of total maize aflatoxin concentrations for participants.

Eating contaminated homegrown maize kernels was the primary risk factor for developing aflatoxicosis. On average, maize samples were collected 33 days (range, 8–112 days) after case-patients’ onset of symptoms. Homegrown maize kernels from case households had significantly higher aflatoxin concentrations than kernels sampled from control households [geometric mean (GM) = 354.5 ppb vs. 44.1 ppb, respectively; *p* = 0.04; Figure 1]. Eating homegrown maize kernels was significantly associated with case status (adjusted OR = 3.0; 95% confidence interval (CI), 1.01–8.8). Owning “bad” homegrown maize kernels (maize with colored flecks, discoloration, unusual odor, or signs of mold) was found to be a risk factor for aflatoxicosis (adjusted OR = 5.9; 95% CI, 1.9–18.2). Case patients who fed their dogs household food reported dog deaths more often (43%) than controls (15%; adjusted OR = 15.2; 95% CI, 1.8–127.4). We did not find an association between case status and the number of portions of maize, beans, or meat participants consumed on a weekly basis.

### Serum aflatoxin B_1_–lysine adduct analysis.

**Figure 2:**
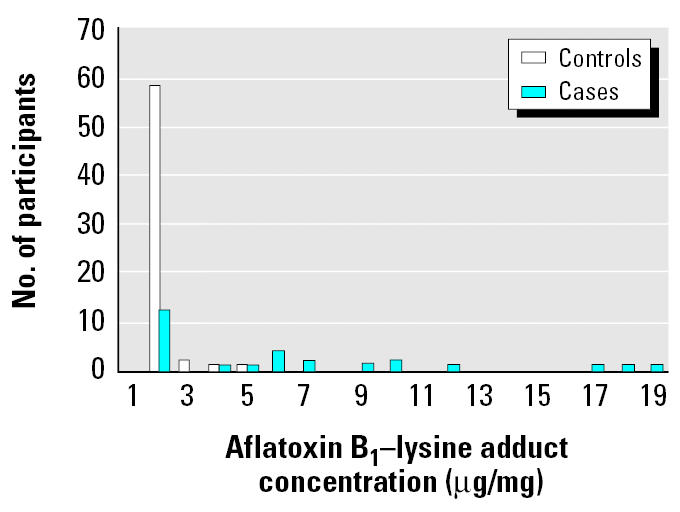
Frequency of serum aflatoxin B_1_–lysine albumin adduct concentrations for participants.

On average, serum samples were collected 33 days after case-patients’ onset of symptoms. Using conditional logistic regression, we found that having aflatoxin B_1_–lysine adduct concentrations at or above the median (0.25 ng/mg) was a risk factor for developing aflatoxicosis (adjusted OR = 14.8; 95% CI, 3.0–72.2). Case patients who provided serum samples (*n* = 29) had higher aflatoxin B_1_–lysine adduct concentrations in their serum than did controls (*n* = 62; GM = 1.2 ng/mg of albumin vs. 0.15 ng/mg of albumin; *p* < 0.001; Figure 2). We found a positive association between concentrations of aflatoxins in homegrown maize and aflatoxin B_1_–lysine adduct concentrations in serum mixed linear regression adjusted for age, sex, village, and district (*p* < 0.05). For each milligram increase in the maize aflatoxin concentration, there was a 0.5 pg/mg increase in the logarithm of the serum aflatoxin B_1_–lysine adduct concentration.

### Serum hepatitis B surface antigen analysis.

There was sufficient serum to analyze 72 (60%) samples for hepatitis B surface antigen. The mean age of participants with positive titers was 33 years, and most of them were female (58%). Eight (44%) of 18 cases had positive titers, while only 4 (7%) of 54 controls had positive titers (Table 2). Using conditional logistic regression, we found that having positive hepatitis B surface antigen titers was a risk factor for acute hepatic failure (adjusted OR = 9.8; 95% CI, 1.5–63.1). When we restricted the conditional logistic regression to participants with negative hepatitis B titers, we found that having aflatoxin B_1_–lysine adduct concentrations at or above the median for this subgroup (0.2 ng/mg) was a risk factor for developing aflatoxicosis (95% CI, 2.1–∞, *p* = 0.004).

### Risk associated with toxin.

Case patients with known dates of death who had provided blood samples (*n* = 8) had higher aflatoxin B_1_–lysine adduct concentrations in their serum than did case patients who survived (*n* = 17; 3.2 ng/mg vs. 0.5 ng/mg; *p* = 0.07) after adjusting for age, sex, and district. In our survival analysis, we found a significant association between time from jaundice to death and serum aflatoxin B_1_–lysine adduct concentration (adjusted hazard ratio = 1.3; 95% CI, 1.04–1.6; *p* = 0.02).

### Risk associated with food preparation and storage.

Storing maize that was not completely dry and storing maize inside the home rather than in a granary were both independently associated with development of aflatoxicosis (OR = 3.5; 95% CI, 1.2–10.3; OR = 12.0; 95% CI, 1.5–95.7, respectively; Table 3). Participants who reported storing their maize mixed with ash had lower concentrations of aflatoxins in their maize than those who did not (GM = 17.4 ppb vs. 142.2 ppb; *p* = 0.05). We did not find an association between case status, the type of container used to store maize (plastic burlap, plastic bucket, woven basket, clay pot, gourd, or sisal), the use of soda and pesticides in the storage area, or the culling of maize kernels that appeared moldy.

## Discussion

### Food consumption and aflatoxin analyses.

This is the first investigation to quantify the association among environmental contamination, a history of exposure, biomarker concentrations, and acute aflatoxicosis. The results of our case–control study suggest that consumption of contaminated maize kernels placed people in this region of Kenya at risk for life-threatening aflatoxicosis (case-fatality rate of 39%). Through systematic sampling of maize and serum from participants, we found a strong association between aflatoxin concentrations in homegrown maize, serum B_1_–albumin adducts, hepatitis B surface antigen titers, and case status.

The aflatoxin concentrations measured from the maize of case patients was comparable with those measured in other acute aflatoxicosis outbreaks. The aflatoxin B_1_–lysine adduct concentrations measured from the serum of case patients are the highest ever reported. This is the first study to quantify aflatoxin B_1_–lysine adduct concentrations in the serum of case patients during an outbreak of acute aflatoxicosis; a critical step in the elucidation of the clinically relevant action levels for aflatoxin exposure. We associated these serum aflatoxin B_1_–lysine adduct concentrations with the risk for life-threatening acute aflatoxicosis.

We found an association between aflatoxin concentrations in maize and aflatoxin B_1_–lysine adduct concentrations in serum from controls. The GM aflatoxin B_1_–lysine adducts concentration in serum from controls is higher than the majority of concentrations documented in population-based studies from countries with a high incidence of liver cancer (Wild et al. 1990). It is unclear why some controls with high aflatoxin B_1_–lysine adduct concentrations did not manifest symptoms of acute hepatitis during the time of the investigation. The concentrations found in controls were not associated with acute symptoms and may have represented chronic exposure to aflatoxins. Chronic exposure to aflatoxins is associated with impaired immunity, malnutrition, and liver cancer (the third most common cause of death from cancer in Africa) (Parkin et al. 2003; Williams et al. 2004). People chronically exposed to elevated concentrations of aflatoxins are three times more likely to develop hepatocellular carcinoma.

We also found an independent association between hepatitis B surface antigen titers and case status. Although people with hepatitis B (which is endemic in Kenya) who are chronically exposed to aflatoxins may be more likely to develop hepatocellular carcinoma, this is the first study to quantify the association between hepatitis B, aflatoxin adducts, and acute hepatitis (Keenlyside et al. 1977; Qian et al. 1994). Further research is needed to determine if the high incidence of liver cancer in eastern Kenya is attributable to chronic asymptomatic exposure to aflatoxins. In addition, clinicians working in areas where aflatoxicosis is endemic should consider obtaining a dietary history for aflatoxin exposure from cases patients with symptoms of acute hepatitis and positive hepatitis B titers.

### Risk factors.

Our case–control study quantified ORs for suspected risk factors described in previous aflatoxicosis outbreaks. As in a 1974 outbreak in India (Krishnamachari et al. 1975b), we found that males were more likely to die from aflatoxicosis, in spite of eating similar quantities of maize as females. We found that acute aflatoxicosis manifests in family clusters, as reported in a 1988 outbreak in Malaysia (Lye et al. 1995). Sharing contaminated food and genetic polymorphisms of cytochrome P_450_ enzymes may place families at risk for aflatoxicosis (Chen et al. 2000). As reported by Ngindu (1982) in a 1981 outbreak in Kenya, we found that, more often than controls, case patients reported dog deaths before developing aflatoxicosis. In the future, reports of deaths in dogs may warn public health officials of a potential aflatoxin contamination of the food supply.

### Food preparation and storage analysis.

Although maize is traditionally stored in granaries, storage inside homes occurs during periods of food shortage; this may have facilitated the contamination of maize with aflatoxins. The rainy season (from March through May) accounts for 80% of annual food production [Food and Agriculture Organization (FAO) 2000]. In 2004, an early and insufficient rainy season caused a food shortage of 156,000 metric tons of maize (Associated Press 2004). Some participants reported storing maize inside their homes to ensure it would not be stolen during the food shortage. Drought conditions stress maize plants and render them susceptible to contamination by *Aspergillus* spp. (Wilson and Payne 1994). The warm environment inside these windowless homes and storage of maize on the dirt floor may have promoted fungal growth in wet maize kernels.

Our case–control study suggests that traditional methods of drying and storing maize in elevated granaries were protective against aflatoxicosis. Traditional granaries are raised structures that are well ventilated, and they promote the drying of grain (FAO 1998). The granaries’ elevated platforms isolate the maize from spores and insects on the ground. We also found that storing maize mixed with ash was associated with lower concentrations of aflatoxin than storing maize without ash. Ash acts as a physical barrier against insects and helps keep maize dry.

### Limitations.

Our case–control study was limited by its retrospective design. It is possible that case patients (or the family members of deceased case patients) may have recalled the amount, source, and quality of maize that was consumed differently than did controls. The aflatoxin concentrations measured in sampled maize may have differed from those consumed by case patients before they became ill with aflatoxicosis. We may not have found an association between the number of portions of maize consumed and case status due to the limited accuracy of the food questionnaires. In addition, it is possible that some case patients developed jaundice as a result of undiagnosed medical conditions unrelated to aflatoxicosis. This potential misclassification would have weakened any demonstrable associations.

## Conclusion

Aflatoxins and other mycotoxins contaminate 25% of agricultural crops worldwide and are a source of morbidity and mortality throughout Africa, Asia, and Latin America (Smith et al. 1994). To prevent future aflatoxicosis outbreaks, it is necessary to explore public health interventions that promote effective production, storage, and processing of homegrown and commercial maize. In addition, surveillance that monitors aflatoxin concentrations in food and incidence of acute jaundice in humans may prevent widespread outbreaks of acute aflatoxicosis (Trucksess and Wood 1994). In the future, serum aflatoxin B_1_ albumin adducts may be used to diagnose acute aflatoxicosis and monitor interventions aimed at reducing aflatoxin exposure (Kensler et al. 1999). Although short-term interventions such as food replacement mitigate the loss of life during outbreaks, it is necessary to develop long-term, culturally appropriate strategies to prevent aflatoxicosis.

## Figures and Tables

**Table 1:** Demographic characteristics [*n* (%)] of jaundiced case patients (*n* = 40) and village controls (*n* = 80), Eastern Province, Kenya, 2004.

Characteristic	Case patients	Controls	*p*-Value^a^
District			
Kitui	21 (52.5)	42 (52.5)	1.00
Makueni	19 (47.5)	38 (47.5)	
Mean age (years)	22.5	26.0	0.37^b^
Age < 15 years	22 (55.0)	31 (38.8)	0.09
Male	25 (62.5)	27 (33.8)	0.003
Family with jaundice	15 (37.5)	3 (3.8)	< 0.001
Heard of outbreak	34 (85.0)	72 (90.0)	0.33

a Values calculated using chi-square test unless indicated otherwise.

b Student’s *t*-test used for comparison of means.

**Table 2:** Serum aflatoxin B_1_–lysine albumin adduct concentration and hepatitis B surface antigen titers (μg/mg of albumin) in cases and controls [GM (*n*)].

	Adduct concentration	
	Cases	Controls
Hepatitis B positive	0.17 (8)	0.08 (4)
Hepatitis B negative	3.55 (10)	0.16 (50)

**Table 3:** Risk factors [*n* (%)] for jaundice among case patients (*n* = 28) and controls (*n* = 43) who ate maize kernels grown on their own farms, Kenya, 2004.

Characteristic	Case patients	Controls	OR (95% CI)
Initial dryness of stored maize			
Wet	15 (53.6)	11 (25.6)	3.5 (1.2–10.3)
Dry	13 (46.4)	32 (74.4)	1.0
Storage location			
House	22 (81.5)	23 (53.5)	12.0 (1.5–95.7)
Granary	5 (18.5)	20 (46.5)	1.0
Preservatives added to storage			
Ash	6 (15.4)	13 (17.6)	1.6 (0.4–5.6)
Insecticide	9 (23.1)	21 (28.1)	0.6 (0.2–1.8)
